# Fabrication of (amino)thiol chelating agents on SBA-15 and MCM-41 and applications in the extraction of Cd(II), Pb(II) and Cr(VI) cations from aqueous solutions

**DOI:** 10.1007/s11356-025-36705-9

**Published:** 2025-07-05

**Authors:** Siphosethu Maqinana, Chrispin B. O. Kowenje, Stephen O. Ojwach

**Affiliations:** 1 School of Chemistry and Physics, University of KwaZulu-Natal, Private Bag X01 Scottsville, Pietermaritzburg, 3209 South Africa; 2 Department of Chemistry, Maseno University, Private Bag, Maseno, Kenya

**Keywords:** Chelating agents, Extraction of metal cations, Functionalization, Kinetics and mechanism, Silica

## Abstract

Reactions of *N*^*1*^*,N*^*3*^-bis(2-mercaptoethyl)isophthalamide with (3-aminopropyl)triethoxysilane (3-APTES) afforded the the corresponding Schiff base chelating ligand, (1Z,3Z)-N^1^,N^3^-bis(2-mercaptoethyl)-N'^1^,N'^3^bis(3(triethoxysilyl)propyl) isophthalimidamide (**L1**) in high yields. Subsequent immobilization of **L1** on mesoporous silica supports (SBA-15 and MCM-41) following a divergent method produced the respective fabricated **L1@SBA-15** and **L1@MCM-41** materials. Characterization of the fabricated chelating agents was accomplished using SEM, TEM, EDX, BET, PXRD, FT-IR, and TGA-DTA/DSC techniques. The fabricated materials were used as adsorbents for the removal of Cd(II), Pb(II) and Cr(VI) metal ions from water. Both the **L1@SBA-15** and **L1@MCM-41** materials demonstrated high extraction efficiencies for Cd(II) and Pb(II) cations, achieving over 98% and 97% removal, respectively, across a broad pH range (3–9). For Cr(VI) anions, relatively lower optimal extraction efficiencies of 67% and 77% were recorded between pH 3–12, for **L1@SBA-15** and **L1@MCM-41**, adsorbents, respectively. Faster extraction kinetics were observed for Cd(II) and Pb(II) cations, with over 94% removal achieved within 30 min, even at low initial metal concentrations (5 ppm) and adsorbent dosages (10 mg). Kinetic modelling showed that the adsorption process followed a *pseudo*-second-order kinetics, with high correlation coefficients (R^2^) for Cd(II), Pb(II), and Cr(VI) of 0.97573, 0.97348, and 0.91017 using **L1@MCM-41**, and 0.9754, 0.97089, and 0.89411 using **L1@SBA-15**, respectively. Equilibrium data best fitted the Freundlich isotherm model, with R^2^ values of 0.97952, 0.99892, and 0.97374 for **L1@SBA-15** and 0.99879, 0.99999, and 0.90923 for **L1@MCM-41**, corresponding to Cd(II), Pb(II), and Cr(VI), respectively. In competitive metal ion systems, the extraction selectivity followed the order Cd(II) > Pb(II) > Cr(VI), with **L1@SBA-15** exhibiting superior overall performance compared to **L1@MCM-41**. The extraction mechanism was postulated to occur through the coordination of the metal ions to the nitrogen/sulfur donor atoms in the chelating agents, consistent with the hard and soft acid and base (HSAB) principle. Furthermore, **L1@MCM-41** demonstrated notable regeneration potential, retaining Cr(VI) extraction efficiencies over four successive cycles.

**Graphical Abstract:**

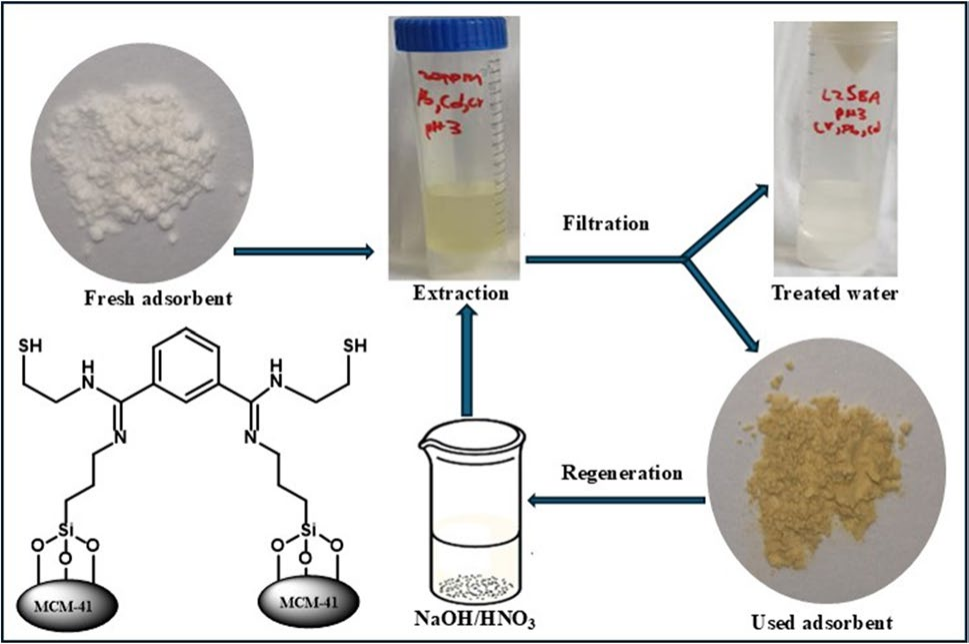

**Supplementary Information:**

The online version contains supplementary material available at 10.1007/s11356-025-36705-9.

## Introduction

The treatment and reuse of wastewater has become a critical global concern due to the presence of several pollutants (Ezzatkhah et al. 2023). Wastewater can be polluted with toxic substances, including heavy metals, due to industrial, agricultural, and natural activities, among other sources (Sheraz et al. 2024). Among these heavy metals, cadmium, lead, and chromium are considered the most hazardous and non-essential due to their extreme toxicities, even at very low concentrations in the body (Zheng et al. 2021; Purrostam et al. 2023). These heavy metal ions also demonstrate high persistence in the environment and thus have the potential to accumulate to levels exceeding permissible limits (Sen 2023). According to the World Health Organization (WHO) and the United States Environmental Protection Agency (USEPA), the permissible concentrations of cadmium, lead, and chromium in drinking water are 0.005, 0.01, and 0.05 ppm, respectively (Dindar et al. 2016). Exceeding these limits can lead to serious health issues, including central nervous system damage, inhibited cell growth, cancer, and reproductive system damage (Dinh Du et al. 2019; Wang et al. 2021).

To this effect, significant efforts have been directed towards removing cadmium, lead and chromium metals from the environment. Conventional remediation methods, such as membrane filtration (Gouda et al. 2023), coagulation/flocculation (Zaki et al. 2023), reverse osmosis (Lumami Kapepula et al. 2022), coprecipitation (Benalia et al. 2022), and ion exchange have been utilized for this purpose. However, these approaches often suffer from key drawbacks such as high operational costs, secondary sludge generation, and inefficiency in the presence of co-contaminants or wide pH fluctuations (Yazdani et al. 2024). Of these techniques, adsorption is widely favoured due to its simple design, low cost, ease of operation, high adsorption rate, and the possible recycling of the adsorbents (Wang et al. 2021; Zhou et al. 2024). To date, various adsorbents, including activated carbon (Abu-Daabes et al. 2023), clay (Teğin et al. 2023), carbon nanotubes (Abdulkareem et al. 2023), polymers (Dewi et al. 2024), chelating resins (Duan et al. 2023), biosorbents (Suganya et al. 2016) and biomass or plant-based materials (Saravanan et al. 2021; Wu et al. 2023) have been employed in heavy metal remediation. However, these adsorbents often suffer from one or a number of drawbacks, such as weak interactions with metal cations and relatively low removal efficiency, thus limiting their applications (Dindar et al. 2015; Lachowicz et al. 2019).

Recent studies on the use of physical adsorbents has focused on mesoporous silica materials such as SBA-15 (Santa Barbara Amorphous) and MCM-41 (Mobil Composition of Matter), modified with chelating organic ligands. This is largely owed to their several desirable features such as tunable pore size, high specific surface area, excellent thermal stability, and ordered microstructure (Pontes Pereira et al. 2015; Dindar et al. 2015; Lachowicz et al. 2019). In addition, compared to other mesoporous adsorbents, SBA-15 offers a significant advantage due to its large and tunable pore size, which facilitates the accommodation of bulky metal complexes and functional groups, thereby enhancing extraction efficiency. Conversely, MCM-41 provides an exceptionally high surface area and a large number of accessible active sites, making it particularly effective for the adsorption of trace-level metal ions. Both SBA-15 and MCM-41 possess a well-ordered hexagonal mesostructure, which promotes efficient diffusion of metal ions and uniform distribution of functional groups. Moreover, their surfaces can be readily functionalized with a wide range of ligands specifically designed to improve selectivity toward target metal ions (He et al. 2018; Ryu et al. 2019).

Schiff base ligands have been widely used as efficient chelating agents for the removal of a number of heavy metal cations due to their strongly coordinating abilities and the ease of tuning their donor abilities to suit specific metal cations (Dindar et al. 2015; Parambadath et al. 2016). Furthermore, studies have shown that immobilization of the Schiff base ligands on solid supports such as SBA-15 and MCM-41 produces highly effective adsorbents (Wang et al. 2021; Zheng et al. 2021). For example, 3-aminopropyltriethoxysilane was grafted onto MCM-41 and used in the removal of Cd(II) and Pb(II) cations from aqueous solutions, achieving maximum adsorption capacities of 14.08 mg/g and 64.21 mg/g, respectively (Dinh Du et al. 2019). In another study, 5-methyl-2-thiophenecarboxaldehyde was immobilized on SBA-15 grafted with (3-aminopropyl)triethoxysilane for selective removal of Cr(III), Cd(II), and Zn(II) cations from water sources (Parambadath et al. 2016).

Among the heavy metals, Cr(VI) poses distinct environmental and remediation challenges due to its coexistence of two stable oxidation states in aqueous environments, the trivalent chromium [Cr(III)] and the more toxic hexavalent chromium [Cr(VI)]. Cr(VI), commonly found as chromate (CrO_4_^2−^) or dichromate (CrO_7_^2−^), is highly soluble, carcinogenic, and significantly more mobile in water systems, making its removal more difficult compared to Cr(III) (Islam et al. 2023). It has been shown that functionalized adsorbents, particularly those incorporating nitrogen and sulfur donor atoms, can effectively remove Cr(VI) through redox-assisted adsorption mechanisms, including electrostatic attraction and reduction to Cr(III) (Li et al. 2021). For example, Fellenz et al. (2017) demonstrated that mesoporous silica MCM-41, functionalized with aminopropyl groups, achieved a Cr(VI) adsorption capacity of 86.4 mg/g. The removal occurred via electrostatic interactions between the negatively charged HCrO_4_^−^ species and positively charged surface ammonium groups, followed by partial reduction of Cr(VI) to Cr(III). The proposed mechanism suggests that this reduction is accompanied by proton release from the adsorbent surface into the solution (Fellenz et al. 2017).

While the synthesis of N^1^,N^3^-bis(2-mercaptoethyl)isophthalamide ligand for removal of mercury from water has been previously described by Atwood and co-workers (Blue et al. 2010), this work extends on its synthesis and application by further modifying this ligand on silane coupling agent, 3-APTES and additional grafting on SBA-15 and MCM-41 through a convergent approach for extraction of Cd(II), Pb(II) and Cr(VI). To the best of our knowledge, the use of *(*(1Z,3Z)-N^1^,N^3^-bis(2-mercaptoethyl)-N'^1^,N'^3^bis(3(triethoxysilyl)propyl) isophthalimidamide immobilized on SBA-15 and MCM-41 as chelating agents synthesized through a convergent approach has not been previously reported for the extraction of Cd(II), Pb(II), and Cr(VI) cations.

Following these promising developments in the design of organic–inorganic hybrid materials for effective heavy metal ion removal, the current study aims to design chelating agents which are effective, selective and recyclable in the removal of Cd(II), Pb(II), and Cr(VI) ions from water. The fabrication of the organic chelating agent on mesoporous silica (SABA-15 or MCM-41) was to enhance the physical separation and reuse of the adsorbents. The fabricated chelating agents were characterized by SEM, TEM, EDX, BET, PXRD, and TGA-DTA/DSC to establish the functional groups present, particle size and morphology, elemental composition, surface area and porosity as well as crystallinity of the materials. The effects of the extraction parameters such as pH, contact time, chelating agent dosage and initial metal concentrations were investigated and are discussed together with detailed studies of the kinetics and mechanisms of extractions.

## Experimental and methodology

### General materials and instrumentation

The reagents: cysteamine chloride, Isophthaloyl chloride (99%), triethylamine (99%), (3-aminopropyl) triethoxy silane (98%), potassium dichromate (K_2_Cr_2_O_7_), cadmium nitrate (Cd(NO_3_)_2_, lead nitrate (Pb(NO_3_)_2_, sodium hydroxide pellets (NaOH), and nitric acid (HNO_3_), were bought from Sigma-Aldrich and used without further purification. The solvents, chloroform, and toluene were purchased from Merck and distilled and dried in molecular sieves before use. NMR spectra were recorded on a Bruker Ultrashield 400 spectrometer, while infrared spectra were recorded on a Perkin-Elmer spectrum 100 range 4000–400 cm^−1^. Scanning electron microscope (SEM) and transmission electron microscope (TEM) were acquired on a ZEISS EVO LS15 and JEOL JEM 1400, respectively. Powder X-ray diffraction (PXRD) patterns were recorded on an X-ray diffractometer (Bruker D8 Advance), while Energy Dispersive X-ray (EDX) analysis was carried out using an Oxford-made EDX detector. Following the Brunauer, Emmett, Teller (BET), and Barrett, Joyner, Halenda (BJH) methods, data was evaluated using BELmaster software version 7.3.2.0. Magnetic susceptibility measurements were performed using a Quantum Design MPMS3 Evercool SQUID magnetometer with a 7 Tesla magnet at 300 K, with variable applied DC magnetic fields ranging from −4 T (−40,000 Oe) to 4 T (40,000 Oe) on a DC mode. Thermal analyses (TGA) and differential scanning calorimetry (DSC) were run on a PerkinElmer Thermogravimetric Analyser 4000 (TGA) and PerkinElmer DSC 4000, respectively. The samples were heated from 25 °C to 800 °C at a 20 °C/min rate under N_2_ atmosphere. The concentration of heavy metal cations in the solution was measured using a Shimadzu Plasma Atomic Emission Spectrometer (ICPE-9820).

## Results and discussion

### Synthesis and characterization of chelating agents

**Scheme 1:**
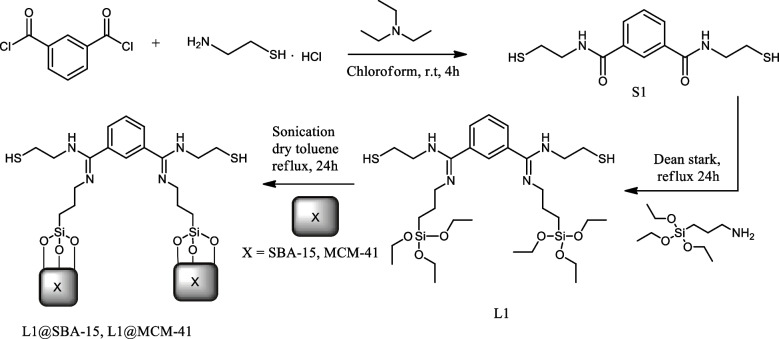
Synthesis and immobilization of **L1** on **SBA-15** and **MCM-41**

The organic ligand *N*^*1*^*,N*^*3*^-bis(2-mercaptoethyl)isophthalamide (**S1**) was synthesized by adopting literature methods in high yields, as shown in Scheme 1 (Blue et al. 2010; Bandara 2022). Subsequent condensation of **S1** with (3-aminopropyl)triethoxysilane (APTES) afforded the corresponding ligand (1Z,3Z)-N^1^,N^3^-bis(2-mercaptoethyl)-N'^1^,N'^3^bis(3(triethoxysilyl)propyl) isophthalimidamide (**L1**) as previously reported (Jayamani et al. 2019). Immobilization of **L1** on **SBA-15** and **MCM-41**,following reported protocols (Akiri and Ojwach 2019), furnished the respective composite materials **L1@SBA-15** and **L1@MCMC-41** in moderate yields as outlined in Scheme 1.

The structural analyses of the organic ligand (**L1**) and its respective fabricated materials were achieved using ^1^H NMR and FT-IR spectroscopies (Figs. S1-S4). The identity of **S1** was confirmed by the presence of the N–H signal at 7.95 ppm (Fig. S2), while the signal at 0.57 ppm was assigned to the aliphatic CH_2_ protons and validated the formation of Schiff base compound **L1** (Fig. S4). The FT-IR spectra of compounds **S1** and **L1** (Fig. S1) exhibited signature *v*_(N–H)_ stretching bands at 3282 cm⁻^1^ and 3296 cm⁻^1^, *v*_(S–H)_ stretching bands at 2531 cm⁻^1^ and 2543 cm⁻^1^ (Fig. S1). More importantly, the signal at 1527 cm^−1^ assigned to the *v*_(C=O)_ stretching band observed in **S1** was absent in **L1**, consistent with the Schiff base condensation to form the imine functionality later. Indeed, the typical *v*_(C=N)_ band was recorded at 1534 cm⁻^1^ in **L1** (Fig. S3). Successful immobilization of **L1** on SBA-15 and MCM-41 supports to form the composite materials **L1@SBA-15** and **L1@MCM-41**, respectively, was deduced from the presence of the *v*_(Si–OH)_ bands at around 800 cm⁻^1^ in both materials (Figs. S5 and S6).

**Fig. 1:**
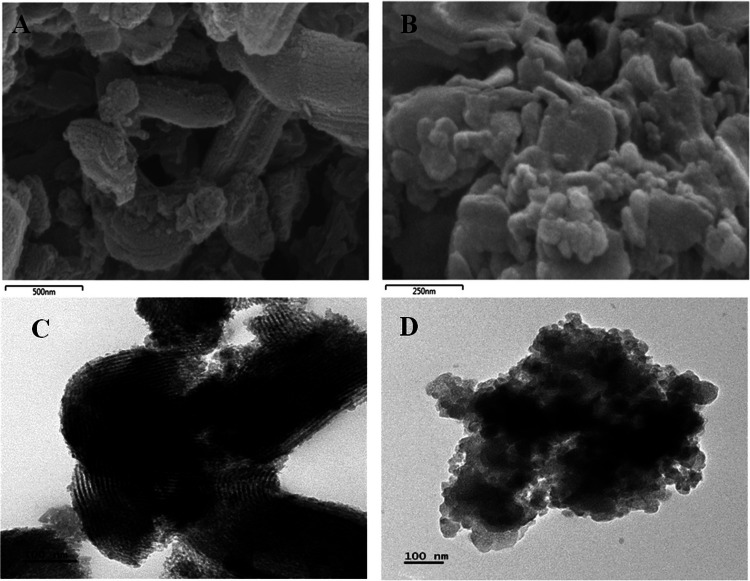
SEM images of (**A**)** L1@SBA-15, **(**B**)** L1@MCM-41,** and TEM images (**C**)** L1@SBA-15, D L1@MCM-41** of the immobilized ligand

The surface morphology of the fabricated ligand was characterized using TEM and SEM, as shown in Fig. 1. The SEM images of **L1@SBA-15** (Fig. 1a and b) displayed rod-like and aggregated particles, while the TEM images of **L1@SBA-15** (Fig. 1c and d) revealed rod-like structures with ordered hexagonal voids. The TEM images of **L1@MCM-14** (Fig. 1d) exhibited quasi-spherical particles, consistent with the agglomerated quasi-spherical morphology observed in the SEM images (Fig. 1b). The dark spots in the composite materials indicated the incorporation of the ligands into the silica matrices. The average nanoparticle sizes of **L1@SBA-15** and **L1@MCM-41**, measured from TEM images using ImageJ software, were 2.03 ± 0.11 nm and 2.61 ± 0.09 nm, respectively (Fig. S7).

The composition and elemental distribution of the silica-immobilized chelating agents were analyzed using EDX and EDX mapping (Fig. S8). The presence of Si and N, O, and S atoms (from the organic ligands) confirmed the successful incorporation of the organic ligand on the SBA-15 and MCM-41 matrices. The measured quantities of N and S donor atoms were 2.4 wt.% and 14.57 wt.% for **L1@SBA-15** and 6.18 wt.% and 4.81 wt.% for **L1@MCM-41**, respectively. Additionally, EDX mapping demonstrated that the elements were uniformly distributed across the surface of the composite materials.

The nitrogen adsorption–desorption isotherms and pore size distributions of the immobilised chelating materials were obtained through BET and BJH analyses (Fig. S9). Both materials **L1@SBA-15** and **L1@MCM-41** exhibited type IV isotherms with H1 hysteresis loops, indicating the presence of mesoporous materials with capillary condensations (Wang et al. 2021; Liang et al. 2023). While in **L1@SBA-15**, a distinct capillary condensation/evaporation step was observed in the P/P_0_ range of 0.6–0.8 (Fig. S9a), **L1@MCM-41** showed a partial evaporation step in the P/P_0_ range of 0.9–1.0 with a decrease in nitrogen adsorption, suggesting that organic ligand significantly blocked the MCM-41 pores (Fig. S9b). These results were corroborated by the larger surface area of 141 m^2^/g and an average pore diameter of 9 nm recorded for **L1@SBA-15**, compared to a much smaller surface area of 7 m^2^/g. recorded for **L1@MCM-41** material. Similar findings exist in literature where the SBA-15 functionalized materials showed an average pore diameter of 8.7 nm (Wu et al. 2020), while the corresponding MCM-41 immobilized composite exhibited much smaller surface areas (Bagheri et al. 2019).

The crystallinity of the immobilized materials was determined using powder XRD (Fig. S10). The powder XRD patterns of **L1@SBA-15** showed typical diffraction peaks for SBA-15 functionalized materials, though with a slight shift towards higher angles due to modification at 2θ = 21° and 25°, corresponding to the (100) and (110) planes, respectively (Bagheri et al. 2019; Wu et al. 2020; Wang et al. 2021; Liang et al. 2023). These shifts mirror those reported for similar organic ligands immobilized on SBA-15, where only the (100) and (110) planes were observed (Thirupathi et al. 2023). Similarly, the **L1@MCM-41** material displayed a prominent (100) plane at 2θ = 21°, significantly reducing the (110) plane intensity. This characteristic could be assigned to the higher content of organic components in the MCM-41 matrix, as corroborated by the EDX data (Fig. S8) and previous reports of Liao et al*.* (Liao et al. 2024).

The thermal stability of the silica fabricated **L1** was assessed using TGA-DTA/DSC analysis, as shown in Fig. S11. The thermal decomposition of both fabricated materials proceeded in three distinct weight-loss stages. The initial weight loss recorded for **L1@SBA-15** and **L1@MCM-41** of 7% and 10% occurred between 50 °C and 260 °C, respectively, and were attributed to the loss of adsorbed water molecules and volatile solvents (Liang et al. 2023). This was followed by a significant weight loss of 32% and 39% between 270 °C and 470 °C for **L1@SBA-15** and **L1@MCM-41**, respectively, corresponding to the degradation of organic materials (Wu et al. 2020). Lastly, the weight losses, observed between 500 °C and 800 °C, of 49% and 55% for **L1@SBA-15** and **L1@MCM-41**, respectively, were attributed to the decomposition of the inorganic mesoporous silica framework. Comparatively, **L1@MCM-41** exhibited higher weight loss compared to **L1@SBA-15**. This points to the high organic matter incorporated in the MCM-41 core, consistent with the BET and EDX data and previous reports of Rabiee and co-workers (Bagheri et al. 2019).

## Extraction of Cd(II), Cr(VI) and Pb(II) ions from aqueous solutions

### The effect of pH on the extractions of the metal cations

**Fig. 2:**
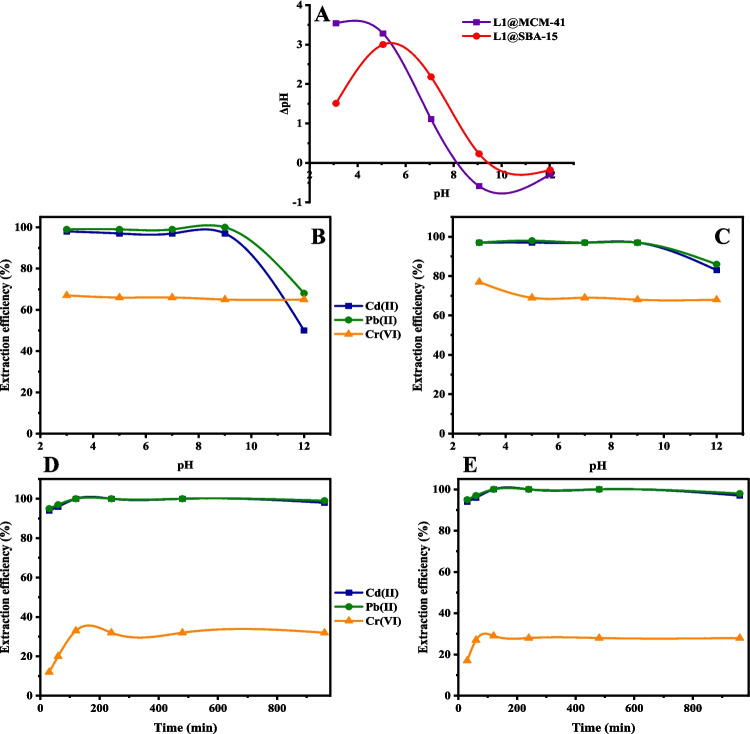
Point of zero charges of fabricated chelating agents **L1@SBA-15** and **L1@MCM-41** (**A**); extraction efficiencies of metal ions on 20 ppm metal solution at different pH values using 0.02 g for 24 h, of **L1@SBA-15 **(**B**) and **L1@MCM-41 **(**C**); extraction efficiencies at different time intervals using 0.02 g using of **L1@SBA-15 **(**D**) and **L1@MCM-41 **(**E**) at pH 3 for Cr(VI), Cd(II) and 5 for Pb(II) cations on 20 ppm solutions

The fabricated materials, **L1@SBA-15** and **L1@MCM-41**, were then applied as adsorbents in the extraction of Cd(II), Pb(II), and Cr(VI) metal ions from aqueous solutions under varied conditions. It is known that the pH of the solution plays a crucial role in the extraction of metal cations, as it influences the surface charges of the adsorbents, the speciation and ionization of the metal ions in the solution (Omotunde et al. 2018) and the mechanism of extractions (Tang et al. 2018). We thus first determined the point of zero charges (pHpzc) of the two adsorbents, as shown in Fig. 2a. The extractions of the metal cations were also studied under different pH values (Fig. 2B and 2 C). The pHpzc values of adsorbents **L1@SBA-15** and **L1@MCM-41** were determined as 9.5 and 8.1, respectively (Fig. 2a). For positively charged metal cations like Pb(II) and Cd(II) extraction efficiency should be favoured at pH values higher than the pHpzc (Omotunde et al. 2018), while for Cr(VI) species, the extraction efficiency is expected to be higher at pH values (Li et al. 2019b; Liang et al. 2023).

The extraction studies under different pH showed that extraction efficiencies for Pb(II) and Cd(II) cations exceeded 96% for both adsorbents, **L1@SBA-15** and **L1@MCM-41**, across a pH range of 3 to 9 (Fig. 2B and 2 C). This observation follows the trends previously reported using MCM-41 functionalized (amino)/thiol chelating agents, which display high extraction efficiencies for Cd(II) and Pb(II) cations at low pH levels within 2–4 (Tang et al. 2018). At pH values higher than 9, the extraction efficiencies for Cd(II) and Pb(II) significantly decreased, possibly due to the precipitation of the metal ions in the solution (Bagheri et al. 2019; Tang et al. 2022). On the other hand, the extraction efficiencies for Cr(VI) anions, using both chelating agents, decreased at pH values higher than the pHpzc (Fig. 2B and 2 C). This trend is expected and could be assigned to the positively charged surfaces at lower pH, thus enhancing electrostatic attraction of the negatively charged Cr(VI) species (Fakhfakh et al. 2024). It has also been shown that the (amino) thiol-based chelating agents could play a bi-functional role, where the amine groups reduce Cr(VI) to Cr(III), which are then bound to the thiol groups (Zaitseva et al. 2013; Li et al. 2023b). Using chelating agent **L1@SBA-15**, optimal pH values of 3 pH for Cr(VI) and 9 for Cd(II) and Pb(II) cations were obtained with extraction efficiencies of 67%, 98%, and 100%, respectively (Fig. 2B). On the hand, using **L1@MCM-41**, the optimal pH values obtained for the Cr(VI) anions and Cd(II) cations were 3 and 5 for Pb(II) cations, achieving extraction efficiencies of 77%, 97%, and 98%, respectively (Fig. 2C).

### The effect of contact time and adsorption kinetics

The extraction of Cd(II), Pb(II), and Cr(VI) ions using the fabricated chelating agents **L1@SBA-15** and **L1@MCM-41** was performed over a time range of 30 to 960 min to determine the optimum contact times (Fig. 2). From the results obtained, both chelating agents showed rapid extractions within 30 min to achieve extraction efficiencies 93% for Cd(II) and Pb(II) cations. In contrast, lower extraction efficiencies for the Cr(VI) anions of 33% and 29% were observed for **L1@SBA-15** and **L1@MCM-41**, respectively, within 120 min (Figs. 2D and 2E). (Ahmed et al. 2020; Waly et al. 2021). Previous related studies reported optimal extraction times of 120 min for Pb(II) cations and Cr(VI) anions using silica-immobilized chelating agents (Ahmed et al. 2020; Waly et al. 2021).

**Fig. 3:**
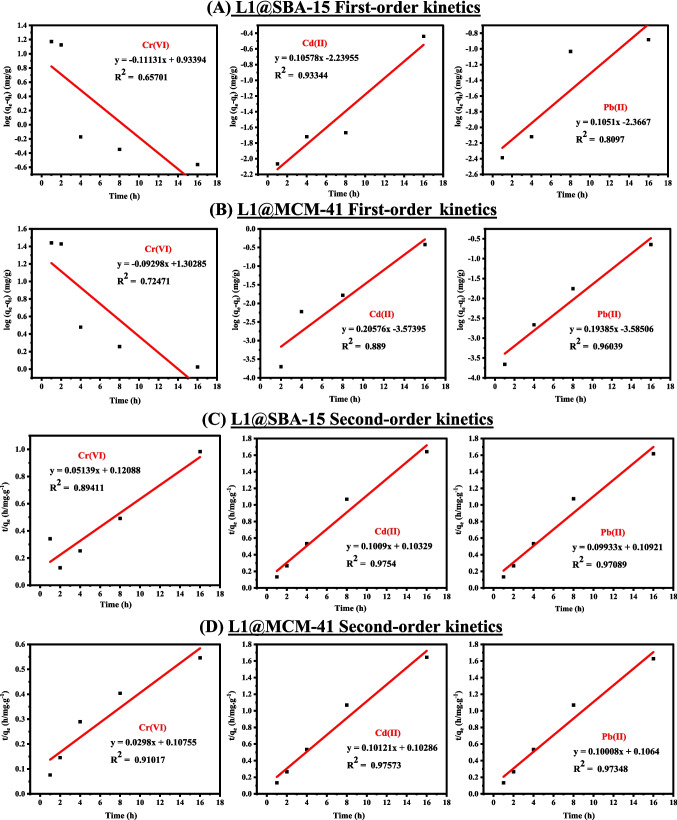
Pseudo-first-order for (**A**)** L1@SBA-15**, and (**B**)** L1@MCM-41** and *pseudo*-second-order (**C**)** L1@SBA-15**, and (**D**)** L1@MCM-41**

The adsorption kinetic data were further elucidated using *pseudo*-first-order and *pseudo*-second-order models. The kinetic models and their parameters are provided in Table S1, while linear fits of the models and the parameters that determine the extraction mechanisms are detailed in Fig. 3 and Table S2**,** respectively. The results indicate that the extractions of Cd(II), Pb(II), and Cr(VI) ions better fitted the *pseudo*-second-order kinetic model, with high correlation coefficients of 0.9754, 0.97089, and 0.89411 for **L1@SBA-15**, and 0.97573, 0.97348, and 0.91017 for **L1@MCM-41**, respectively, compared to the *pseudo*-first-order model. Moreover, the theoretical q_e_​ values calculated for Cd(II), Pb(II), and Cr(VI) ions were 9.91080, 10.0674, and 19.4590 for **L1@SBA-15**, and 9.88044, 9.99200, and 33.5570 for **L1@MCM-41** (Table S2). These values closely matched the experimental q_e_ values for the *pseudo*-second-order model, indicating better agreement than with the *pseudo*-first-order model. The kinetics data thus suggest that the extraction of the metal cations occurred primarily through chemisorption, involving the formation of coordination bonds (Lachowicz et al. 2019).

### The effect of initial concentration and adsorption isotherms

**Fig. 4:**
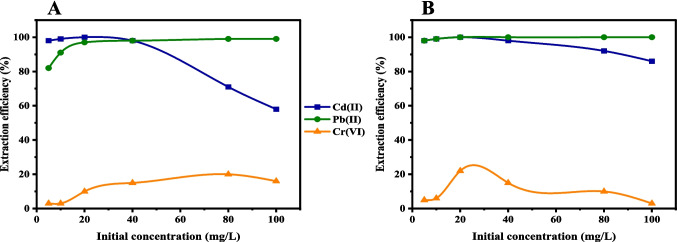
Extraction efficiency at different initial concentrations using 0.02 g of (**A**)** L1@SBA-15** and (**B**)** L1@MCM-41** at pH 3 for Cr(VI), Cd(II) and 5 for Pb(II) for 2 h

The effect of the initial concentration of the metal cations on their extraction efficiencies was studied using both chelating agents **L1@SBA-15** and **L1@MCM-41** from 5 to 100 mg/L (Fig. 4). Using a chelating agent, **L1@SBA-15**, the extraction efficiencies of the metal cations increased with increasing initial concentrations of the metal salts solutions attaining an optimum dosage of 40 mg/L and 80 mg/L for Cd(II) cations and Cr(VI) anions respectively (Fig. 4). This is explained from the available binding sites on the surface of the chelating agents at lower metal concentrations (Tighadouini et al. 2022). However, beyond 40 mg/L for Cd(II) cations and 80 mg/L for Cr(VI) anions, the extraction efficiencies decreased as the initial concentration increased using **L1@SBA-15** adsorbent. Similarly, using **L1@MCM-41**, extraction efficiencies for Cd(II) cations and Cr(VI) anions were recorded at maximum dosages of 80 mg/L and 20 mg/L, respectively. Similar findings have been reported for Cr(VI) anions extraction, where the removal efficiency decreased once the initial concentration of the solution reached 100 mg/L using MCM-48-SH adsorbent (Li et al. 2023a). Interestingly, the extraction of Pb(II) cations by both chelating agents was insignificant over the studied concentrations. While this behaviour is unclear to us at this stage, it is evident that both chelating agents have high adsorption capacities for Pb(II) cations.

**Fig. 5:**
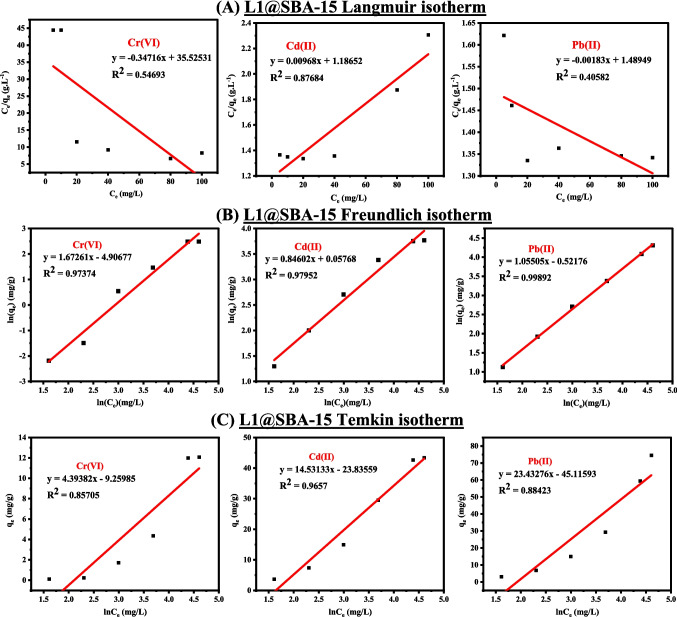
Extraction of metal cations using **L1@SBA-15** fitted on (**A**) Langmuir, **B** Freundlich, and (**C**) Temkin adsorption isotherms

The adsorption isotherms were analysed using the Langmuir, Freundlich, and Temkin models to shed light on the nature of the metal cations'adsorption on the chelating agents'surface. The interpretations of the isotherms and their associated parameters are summarized in Table S3, while linear fits of the isotherms of **L1@SBA-51** and **L1@MCM-41** are presented in Figs. 5, and S12, respectively. The calculated parameter values are provided in Table S4. The linear fits of the isotherms revealed that the extraction of Cd(II), Pb(II), and Cr(VI) ions followed the Freundlich model, with high correlation coefficients of 0.97952, 0.99892, and 0.97374 for **L1@SBA-15** and 0.99879, 0.99999, and 0.90923 for **L1@MCM-41**, respectively, compared to the Langmuir and Temkin models. This suggests that the extraction of metal cations occurred through a heterogeneous and multilayer adsorption process (Dobrzyńska 2021; Tang et al. 2022). The Freundlich constants, *n,* and *K*_*f*_, are used to measure the adsorption capacity of a given material, where *n* > 1 suggests favourable extraction and *n* = 1 indicates linear adsorption (Parambadath et al. 2016; Suhail et al. 2020). The calculated *n* values within the range of 0.80–1.18 (Table S4) demonstrated that **L1@SBA-15** and **L1@MCM-41** displayed favourable extraction affinities for metal ions in the following order: Cd(II) > Pb(II) > Cr(VI).

### The effect of chelating agent dosage

**Fig. 6:**
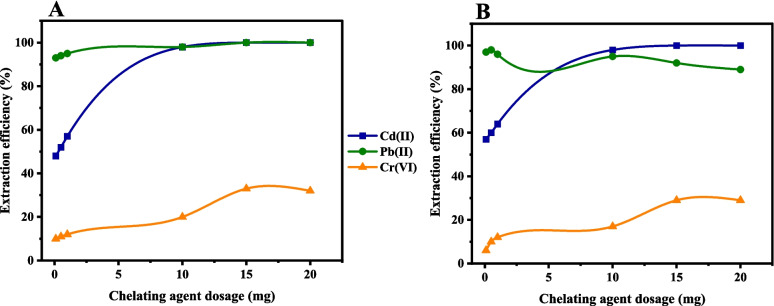
The extraction efficiency at different dosages using (**A**)** L1@SBA-15** and (**B**)** L1@MCM-41** at pH 3 for Cr(VI), Cd(II), and 5 for Pb(II) ions on 20 ppm solution for 2 h

The effect of the chelating agent dosage on the extraction efficiencies of Cd(II), Pb(II), and Cr(VI) ions was investigated by varying the adsorbent from 0.10 to 20 mg (Fig. 6). The results showed that the extraction efficiencies of these metal cations increased with increase in adsorbent dosage up to an optimum value, depending on the adsorbent and the metal cation. For example, using **L1@SBA-15**, a dosage of 15 mg was recorded as the optimum corresponding extraction efficiencies of > 99% for Cd(II) and Pb(II) and 33% for Cr(VI) ions (Fig. 6a). A further increase in the dosage to 20 mg, did not alter the extraction efficiencies of the metal cations. The chelating agent **L1@MCM-41** displayed comparable trends for Cd(II) and Cr(VI), ions to record an optimum dosage of 15 mg, corresponding to extraction efficiencies of > 99% and 29% for Cd(II) and Cr(VI) ions respectively (Fig. 6b). However, using **L1@MCM-41,** the optimum dosage for Pb(II) cations was observed at a much lower value of 0.5 mg, achieving 98% extraction efficiency (Fig. 6b). This unique trend for Pb(II) cations is consistent with the data discussed for the initial metal concentrations (Fig. 4) and points to higher adsorption capacities of these chelating agents for Pb(II) cations (Pandey et al. 2023). The reduction of extraction efficiencies of the metal ions above the adsorbent dosages of 15 mg for Cd(II) and Cr(VI) and 20 mg for Pb(II) ions has been reported and largely attributed to the aggregation of the chelating agents thus limiting the number of available binding sites (Alqadami et al. 2017).

### Competitive and selective extraction of the metal cations

**Fig. 7:**
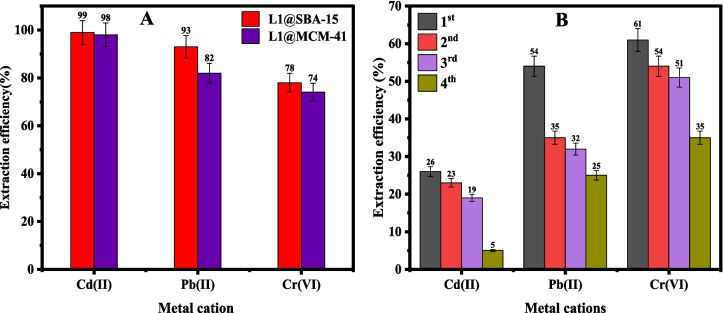
**A** Extraction efficiency using 0.02 g of chelating agents on 20 ppm mixed metal cations solution at pH 3 for 2 h and (**B**) regeneration cycles and re-use of **L1@MCM-41**

The selective extraction of the three metal cations by the chelating agents **L1@SBA-15** and **L1@MCM-41** was assessed using a 20-ppm mixed metal cation solution at pH 3 for 2 h (Fig. 7a). The results indicated that both the chelating agents **L1@SBA-15** and **L1@MCM-41** showed selectivity towards the metal cations in the order Cd(II) > Pb(II) > Cr(VI). The higher extractions of the soft Cd(II) cations, coupled with the lower affinities towards the hard Cr(VI) anions, can be explained by the Hard-Soft Acid–Base (HSAB) theory (Shen et al. 2018) and agree with the presence of the soft S-donor atoms in the organic motif (Prabha Padinhattath and Gardas 2025). Similarly, the high preference for moderate Pb(II) cations, could be derived from the presence of the moderate N-donor in the ligand framework. Additionally, the chelating agents demonstrated similar extraction behaviours in both single-metal and mixed-metal solutions under identical experimental conditions. For example, **L1@SBA-15** achieved extraction efficiencies greater than 99% for Cd(II) and Pb(II) cations in single-metal solutions, which were comparable to efficiencies of 99% and 93%, respectively, observed in mixed-metal systems. Notably, Cr(VI) anions showed an improved extraction efficiency of 78% in the mixed-metal solution compared to 66% in the single-metal system. These results suggest potential synergistic effects in multi-metal systems and highlight the robustness of the chelating agents. The nature of the silica support was also found to influence the extraction efficiencies of the chelating agents. In general, the SBA-15 functionalized adsorbent, **L1@SBA-15,** displayed slightly higher extraction efficiencies than the MCM-41 immobilized material, **L1@MCM-41,** across all the metal cations. To illustrate this observation, extraction efficiencies of 82% and 93% for Pb(II) cations were reported for **L1@MCM-41** and **L1@SBA-15,** respectively (Fig. 7a)**.** This trend can be explained by the higher surface area and pore diameter of 141 m2/g ad 9 nm respectively for **L1@SBA-15** compared to a much lower surface of 7 m2/g, exhibited by **L1@MCM-41**, in good agreement with previous findings of Ali et al*.* (Ali et al. 2016).

### Regeneration and reuse of the chelating agent

The primary aim of immobilizing the studied organic chelating agent on SBA-15 and MCM-41 supports was to enable efficient separation and reuse of the adsorbents. We thus carried out the regeneration and reuse of the chelating agent **L1@MCM-41** using 1.0 M HNO_3_, followed by re-extraction of the metal cations from a 20 ppm solution at their optimal pH conditions (Fig. 7b). While the recycling experiments for the Cd(II) and Pb(II) cations registered significant drops, the recycling experiments of the Cr(VI) anions were quite promising. For example, while Cd(II) cations registered a drop from 99 to 26% in the initial and first recycling experiments, Cr(VI) anions registered 75% and 63% in the initial and first recycling extractions, respectively (Fig. 7). Indeed, by the fourth recycling experiments, Cd(II) cations had negligible extraction efficiency affording only 5% compared to 35% displayed for Cr(VI) anions). This reduction in efficiency is attributed to the loss of chelating agent mass during the stripping and washing steps of each subsequent cycle. A similar trend was observed in the recycling experiments in the removal of Cr(VI) anions and Pb(II) cations, with declines in extraction efficiencies from 73 to 35% and 81% to 24% in the first and fourth cycles being reported, respectively (Bayuo et al. 2020). While losses in the adsorbent during the recycling experiments may account for the decline in extraction efficiencies, the differences in the extraction efficiencies observed for Cd(II) cations and Cr(VI) anions also point to some chemical changes to the adsorbent.

### Mechanism of extraction and coordination chemistry

The adsorption mechanism and the nature of the metal–ligand interactions involved in the extraction of Cd(II), Pb(II), and Cr(VI) metals were elucidated using FT-IR and ^1^H NMR spectroscopies. Comparison of the FT-IR spectra of the fresh and used fabricated chelating agents (Fig. S13) revealed significant spectral changes upon metal adsorption. Specifically, the disappearance of the ν(S–H) stretching bands at 2119 cm^−1^ and 2117 cm^−1^ in **L1@SBA-15** and **L1@MCM-41**, respectively, indicated the deprotonation of the thiol group, suggesting its involvement in metal coordination. The observed shifts in the N–H stretching bands from 3299 cm^−1^ to 3291 cm^−1^ in **L1@SBA-15** further supported the participation of the amine functionality in the coordination sphere of the metals. These observations are consistent with the formation of coordination complexes, where the metal ions interact directly with the N and S donor atoms of the ligand *via* coordinate covalent bonding, as proposed in Scheme S1. The simultaneous disappearance of the S–H band and shift in the N–H band suggest a bidentate coordination mode through both sulfur and nitrogen atoms. To further validate this coordination mechanism, the Cd(II) complex of the unanchored proligand **L1** was independently synthesized and characterized. The FT-IR spectrum of the isolated Cd(II) complex closely resembled that of the used chelating agents, with the complete loss of the ν(S–H) band at 2061 cm^−1^ (Fig. S14), reaffirming thiol deprotonation and coordination. The ^1^H NMR spectrum provided additional insights: the S–H proton signal present at 0.98 ppm in the free ligand (Fig. S15a) was absent in the Cd(II) complex (Fig. S15b), while the N–H proton signal remained but exhibited a slight shift, appearing at a similar chemical shift (2.03 ppm). This spectral pattern indicates that while the thiol group undergoes deprotonation and binds directly to the metal centre, the amine group is either directly involved in coordination or stabilized through hydrogen bonding or electron donation to the metal ion. The combined FT-IR and NMR data confirm the formation of chelated metal–ligand complexes, likely involving the generation of monoanionic thiolate ligands. The proposed complex, [**Cd**_**2**_**(L1)**_**2**_], as illustrated in Scheme S2, consists of two Cd(II) ions bridged by two **L1** ligands through S and N donor atoms. This chelation not only stabilizes the complex but also enhances the selective binding of soft and borderline acid cations like Cd(II) and Pb(II), consistent with the observed extraction trend. Therefore, the mechanism of adsorption can be primarily attributed to specific chemical interactions (chemisorption) involving ligand deprotonation and coordinate bonding, particularly through thiol and amine functionalities, leading to the formation of stable metal complexes. These interactions account for the high affinity, selectivity, and rapid adsorption kinetics observed in the study.

### Comparisons of current chelating agents with reported systems

The extraction capacities of the current fabricated chelating agents **L1@SBA-15** and **L1@MCM-41** were compared with similar commercial and synthesized adsorbents reported in the literature (Table 1). Compared to the commercial granular activated carbon (CG AC, 16–50 mesh, purchased from Indo German, product code wt.-c830), the adsorbents synthesized in this study exhibited Cd(II) and Pb(II) cations adsorption capacities ranging from 43.35 to 74.91 mg/g, whereas the commercial CG AC demonstrated higher capacities of 94.43 to 97.63 mg/g for the same metal ions (Table 1, **entries 1 vs 8–9**). However, the adsorption capacities reported in this study were achieved under more favourable experimental conditions, specifically using a significantly lower adsorbent dosage of 0.02 g and an initial metal ion concentration of 100 mg/L, in contrast to the CG AC conditions, which involved 0.25 g dosage and 300 mg/L initial concentration (Table 1, **entries 1 vs 8–9**). In contrast, the adsorption capacities obtained in this work (43.35–74.91 mg/g) were comparable to those reported for another commercial adsorbent, NTA-silica gel (cationic silica gel, product code 19613-168B, Aladdin Industrial Corporation), which exhibited capacities ranging from 53.14 to 76.22 mg/g for Cd(II) and Pb(II) (Table 1, **entries 2 vs 8–9**). Notably, the performance of NTA-silica gel was recorded after a significantly longer contact time of 1440 min using a 0.01 g dosage. In contrast, the synthesized adsorbents in this study reached comparable capacities within just 120 min, albeit with a higher adsorbent dosage of 0.02 g (Table 1, **entries 2 vs 8–9**). In comparison to similar reported adsorbents, the chelating agents **L1@SBA-15** and **L1@MCM-41** demonstrated fast and relatively high extraction capacities for Cd(II) and Pb(II) cations under both acidic and basic conditions. For example, **L1@SBA-15** and **L1@MCM-41** recorded extraction capacities ranging from 43.35 to 74.91 mg/g to for both Cd(II) and Pb(II) cations within 120 min at pH 3–9 with dosages of 0.02 g, compared to the MCM-41 supported amino ligand described by Dinh et al. (2019) only display extraction capacity of 14.08 mg/g for Cd(II) at pH 9 after 240 min with higher dosage of 0.05 g (Table 1, **entries 3 vs 8–9**). Additionally, Omotunde et al. (2018) obtained a smaller adsorption capacity of 28.41 mg/g for Cd(II) using a Thiolated silica adsorbent at a relatively higher dosage of 0.025 g after 360 min, compared to 43.35 and 64.28 mg/g obtained using 0.02 g after 120 min (Table 1, **entries 5 vs 8–9**). In the case of Cr(VI) anions, the current chelating agents exhibited smaller extraction capacities between 10.89 to 12. 07 mg/g compared to 26.83 mg/g and 50 mg/g of the previously reported adsorbents under similar experimental conditions, including initial concentration, adsorbent dosage, and contact time (Table 1, **entries 6–7 vs. 8–9**).

**Table 1:** Comparison of chelating agents **L1@SBA-15** and **L1@MCM-41** with reported adsorbents in the extraction of Cd(II), Cr(VI) and Pb(II) metal ions

Entry	Adsorbent^a^	pH	Time (min)	Dosage (g)	Volume (ml)	Initial Conc.(mg/L)	Adsorption capacity (mg/g)	Ref
1	CG AC(16–50 mesh)	7	120	0.25 0.25	50 50	300 300	Cd^2+^: 94.43 Pb^2+^: 97.63	(Kavand et al. 2020)
2	NTA-silica gel	5	1440	0.01 0.01	10 10	100 100	Cd^2+^: 53.14 Pb^2+^: 76.22	(Li et al. 2019a)
3	Aminopropyl-MCM-41	9 6	240 240	0.05 0.05	50 50	70.3 64.7	Cd^2+^: 14.08 Pb^2+^: 64.21	(Dinh Du et al. 2019)
4	DPC-SBA-15	5.75	25	0.045	40	25.39	Cd^2+^: 160	(Danesh-Khorasgani et al. 2021)
5	Thiolated silica	5	360	0.025	20	100	Cd^2+^: 28.41	(Omotunde et al. 2018)
6	Urea-SBA-15	5 4 2.5	120 120 90	0.05 0.05 0.05	N/A N/A N/A	100 100 100	Cd^2+^: 30.53 Pb^2+^: 43.85 Cr^6+^: 26.83	(Ezzatkhah et al. 2023)
7	MS-SBA-15-Melamine	3	360	0.02	25	20	Cr^6+^: 50	(Purrostam et al. 2023)
8	L1@SBA-15	3–9 3–9 3	120 120 120	0.02 0.02 0.02	15 15 15	80 100 100	Cd^2+^: 43.35 Pb^2+^: 74.52 Cr^6+^: 12.07	This work
9	L1@MCM-41	3–9 3–9 3	120 120 120	0.02 0.02 0.02	15 15 15	100 100 100	Cd^2+^: 64.28 Pb^2+^: 74.91 Cr^6+^: 10.89	This work

^**a**^**CG AC(16–50 mesh):** commercial granular activated carbon (16–50 mesh), **NTA-silica gel:** Commercial silica gel (cation silica gel (19,613- 168B) modified with amino groups, **Aminopropyl-MCM-41:** aminopropyl grafted on MCM-41, **DPC-SBA-15:** SBA-15 functionalized on 1,5-diphenyl carbazide, **Thiolated silica:** silica gel functionalized with thiourea, **Urea-SBA-15:** urea amine group functionalized on SBA-15, **MS-SBA-15-Melamine:** SBA-15 grafted on 3-aminopropyl trimethoxy silane and functionalized on melamine. NA-Not identified

## Conclusions

This study reports the successful syntheses and application of a novel mixed N^S-donor chelating ligand (**L1**), functionalized onto mesoporous silica supports SBA-15 and MCM-41, to afford **L1@SBA-15** and **L1@MCM-41** composite adsorbents. Structural characterization of the fabricated materials confirmed the retention of the mesoporous architecture and successful incorporation of the chelating ligand in the silica matrix. The fabricated materials demonstrated excellent adsorption efficiences for Cd(II), Pb(II), and Cr(VI) ions in aqueous media. Adsorption studies revealed high removal efficiencies for Cd(II) and Pb(II) cations, exceeding 99% within 120 min under optimized conditions. The adsorbents displayed selective affinity in competitive metal ion systems, with the extraction efficiencies following the order Cd(II) > Pb(II) > Cr(VI). This trend is consistent with HSAB principle, reflecting the preferential binding of soft Cd(II) and Pb(II) metal cations to the soft donor atoms (N and S) in the ligand. Comparatively, **L1@SBA-15** exhibited superior performance relative to **L1@MCM-41**, likely due to differences in the surface area, pore size distribution, and ligand accessibility. Kinetic modelling showed that the adsorption process followed pseudo-second-order kinetics, suggesting chemisorption as the rate-limiting step. Equilibrium data were better fitted by the Freundlich isotherm model, indicating heterogeneous surface adsorption and multilayer formation. Post-adsorption FT-IR and spectral analyses confirmed the coordination of metal ions to the sulfur and amine nitrogen (N–H) donor atoms, forming stable chelated complexes. These findings validate the effectiveness of **L1@SBA-15** and **L1@MCM-41** chelating agents as recyclable and selective adsorbents for the removal of toxic heavy metals from aqueous environments. The high extraction efficiencies of the adsorbents in mixed-metal solutions, coupled with their regeneration ability, render them as potential chelating agents for extractive metallurgy or the removal of specific metal ions in industrial effluents. This will thus form our future studies to explore the applications of these adsorbents in the purification of industrial effluences and heavy metal polluted natural water bodies and the possibility of industrial application on a large scale.

## Recommendations for future research

One of the future prospects of this research study is evaluation of the performance of these adsorbents in the removal of heavy metals from both natural and industrial wastewaters. Depending on the outcome of these real water studies, pilot studies at larger or industrial scales, which will involve both large scale production and applications of the adsorbents. Another important future study is the toxicity assessments of the treated effluents to identify any potential environmental risks, including the emergence of secondary pollution from the used adsorbents.

## Supplementary Information

Below is the link to the electronic supplementary material.

Supplementary file1 (DOCX 4688 KB)

## Data Availability

All the data associated with this manuscript are included in this article and also available as supplementary materials..

## Declarations

### Clinical trial number

This is not applicable.
