# Supplementary material for: Fabrication of (amino)thiol chelating agents on SBA-15 and MCM-41 and applications in the extraction of Cd(II), Pb(II) and Cr(VI) cations from aqueous solutions
Source: Environ Sci Pollut Res Int. 2025 Jul 5;32(29):17776–91. doi: 10.1007/s11356-025-36705-9 (PMC12325471; doi:10.1007/s11356-025-36705-9)
Supplement: Supplementary file 1 — Supplementary file1 (DOCX 4688 KB) [file 11356_2025_36705_MOESM1_ESM.docx]

**Fabrication of (amino)thiol chelating agents on SBA-15 and MCM-41 and applications in the extraction of Cd(II), Pb(II) and Cr(VI) cations from aqueous solutions**

*Siphosethu Maqinana ^1^, Chrispin B. O. Kowenje,^2^ Stephen O. Ojwach^1^**

*^1^School of Chemistry and Physics, University of KwaZulu-Natal, Pietermaritzburg Campus, Private Bag X01 Scottsville, 3209, South Africa; smaqinana@gmail.com.*

*^3^Department of Chemistry, Maseno University, Private Bag, Maseno, Kenya;* [*ckowenje@maseno.ac.ke*](mailto:ckowenje@maseno.ac.ke)

SUPPLEMENTARY MATERIALS

- 1. *Synthesis* *of* *N^1^,N^3^-bis(2-mercaptoethyl)isophthalamide (****S1****)*

A solution of cysteamine chloride (2.0 g; 25.9 mmol) was dissolved in 10 ml dry chloroform. To this solution, 5 ml dry chloroform containing triethylamine (5.2 g; 51.8 mmol) was added. This mixture was stirred for 1 h under nitrogen gas at room temperature. A solution of Isophthaloyl chloride (1.75 g; 8.84 mmol) was dissolved in 5 ml dry chloroform added to the solution and further stirred for 12 h. A clear violet solution was obtained and washed with 40 ml 10% HCl solution three times. The chloroform layer obtained was dried using MgSO_4_. The solvent was removed using a rotor vapor and the obtained white powder (**S1**) was dried at room temperature for 24 h. Yield = 2.87 g (78%). ^1^H NMR (400 MHz, CDCl_3_) δ ppm: 1.44 (s, 1H, S-H), 2.83 (M, 2H, CH_2_-CH_2_-SH), 3.67 (M, 2H, NH-CH_2_-CH_2_), 7.95 (s, 1H, N-H), 7.5 (d, 1H, Ph), 7.28 (t, 1H, Ph), 8.23 (s, 1H, Ph). IR νmax/ cm^-1^: ν(N-H) = 3282, ν(C-H) = 2977, ν(S-H) = 2531, ν(N-H_bend_) = 1628, ν(C=O) = 1527, ν(C=C) = 1354, ν(C-N) = 1241, ν(C-S) = 687.

- 1. *Synthesis of* *(1Z,3Z)-N^1^,N^3^-bis(2-mercaptoethyl)-N'^1^,N'^3^ bis(3(triethoxysilyl)propyl) isophthalimidamide (****L1****)*

The prepared white powder of **S1** ligand (1.05 g, 3.71 mmol) was dissolved in 15 ml dry toluene under reflux at 100℃. Then, a solution of (3-aminopropyl) triethoxy silane (APTES) (1.64 g, 7.43 mmol) in dry toluene (10 ml) was added, and the mixture was further refluxed for 24 h using Dean-Stark apparatus. At the end of the reaction, the organic solvent was evaporated using a rotor vapor. A white powder **L1** was obtained and dried under a vacuum oven at 60℃ for 4h. Yield: 1.80 g (67%). ^1^H NMR (400 MHz, DMSO-d_6_) δ ppm: 0.57 (t, 2H, CH_2_-Si), 2.97 (t, 9H, -CH_2_-CH_3_), 3.58 (m, 2H, O-CH_2_-CH_3_), 1.43 (m, 2H, C-CH_2_-C), 2.66 (t, 2H, N-CH_2_-C), 8.75 (s, 1H, N-H), 1.06 (s, 1H, S-H), 1.43 (M, 2H, CH_2_-CH_2_-SH), 2.30 (M, 2H, NH-CH_2_-CH_2_), 7.94 (d, 1H, Ph), 7.53 (t, 1H, Ph), 8.31 (s, 1H, Ph), IR νmax/ cm^-1^: ν(N-H) = 3296, ν(C-H) =2969, ν(S-H) = 2543, ν(N-H_bend_) = 1629, ν(C=N) =1534, ν(C=C) =1357, ν(C-N) =1286, ν(Si-O) =1028, ν(C-S) =686.

- 1. *Immobilization of* ***L1*** *on SBA-15 (****L1@SBA-15****)*

To a suspension of SBA-15 (0.25 g) in dry toluene (30 ml), **L1** ligand (0.50 g, 0.72 mmol) was added, and the mixture was then sonicated for 30 min. The reaction mixture was further refluxed at 85°C for 14 h. After the reaction period, the crude product was filtered and washed 3 times with 10 ml of dry toluene. A white solid powder, **L1@SBA-15** chelating agent was subsequently dried in an oven at 65 ℃ for 24 h. Yield = 0.72 g (96%). IR νmax/ cm^-1^: ν(N-H) = 3292, ν(S-H) = 2112, ν(N-H_bend_) = 1637, ν(C=N) = 1537, ν(C=C) = 1475, ν(C-N) = 1289, ν(Si-O) = 1069, ν(Si-OH) =801, ν(C-S) = 690.

- 1. *Immobilization of* ***L1*** *on MCM-41 (****L1@MCM-41****)*

To a suspension of MCM-41 (0.25 g) in dry toluene (30 ml), **L1** ligand (0.50 g, 0.72 mmol) was added, and the mixture was then sonicated for 30 min. The reaction mixture was further refluxed at 85°C for 14 h. After the reaction period, the crude product was filtered and washed 3 times with 10 ml of dry toluene. A white solid powder, **L1@MCM-41** chelating agent, was subsequently dried in an oven at 65 ℃ for 24 h. Yield = 0.73 g (97%). IR νmax/ cm^-1^: ν(N-H) = 3292, ν(S-H) = 2108, ν(N-H_bend_) = 1636, ν(C=N) = 1536, ν(C=C) = 1485, ν(C-N) = 1289, ν(Si-O) = 1058, ν(Si-OH) = 800, ν(C-S) = 689.

- 1. *Determination of the point of zero charge of the chelating agents*

The chelating agents' point of zero charge (pH_PZC_) was determined using the solid addition method (Dang Son et al. 2016). A 0.1 M KCl electrolyte solution was prepared, and 15 mL aliquots were distributed into various conical flasks. The pH of these solutions was adjusted to values ranging from 3 to 12 using 0.1 M NaOH and 0.1 M HCl. The initial pH (pH_i_) of each solution was recorded. Subsequently, 0.1 g of the chelating agents (**L1@SBA-15** and **L1@MCM-41**) were added to each flask, and the mixtures were stirred at room temperature for 24 h. After stirring, the chelating agents were removed from the solutions by centrifugation and filtration, and the final pH (pH_f_) was measured. The change in pH (ΔpH) was calculated and plotted against the pH_i_. The point of zero charge of the chelating agents was identified as where the plot intersects the abscissa at ΔpH = 0.

- 1. *General extraction studies of metal cations*

Stock solutions with a concentration of 1000 mg/L for Cd(II), Cr(VI), and Pb(II) cations were prepared by dissolving 2.74 g of Cd(NO_2_)·4H_2_O, 5.65 g of K_2_Cr_2_O_7_, and 1.60 g of Pb(NO_3_)_2_ in 1 L of deionized water, respectively. Working solutions were prepared by appropriate dilution of these stock solutions. Extraction experiments for the heavy metal cations using **L1@SBA-15** and **L1@MCM-41** chelating agents were conducted based on various studies reported in the literature (El-Sheikh et al. 2019; Shtaiwi et al. 2020; Zhang et al. 2020). Extractions were conducted in triplicate at various initial concentrations, pH levels, contact times, and chelating agent dosages.

- - 1. *The effect of pH on extraction of metal cations*

The pH ranges from 3 to 12 were achieved using 1 M NaOH and 1 M HNO_3_ solutions, and extractions were conducted using 2 mg of **L1@SBA-15** and **L1@MCM-41** for 24 h.

- - 1. *The effect of contact time and adsorption kinetics on the extraction of metal cations*

For adsorption kinetics experiments, the contact time was varied from 15 min to 960 min at optimum pH values of 3 for Cr(VI), Cd(II) and 5 for Pb(II) cations using 15 mL of 20 ppm metal solutions.

- - 1. *The effect of initial concentration and adsorption isotherms on the extraction of metal cations*

For adsorption isotherms, 15 mL of the metal salt solutions' initial concentration varied between 5 mg/L and 100 mg/L at optimum pH 3 for Cr(VI), Cd(II) and 5 for Pb(II) cations for 2 h.

- - 1. *The effect of chelating agents’ dosage on the extraction of metal cations*

Extraction experiments with varying chelating agent dosages were conducted using 0.10 mg to 20 mg of the adsorbent on 20 ppm solution with pH 3 for Cr(VI), Cd(II) and 5 for Pb(II) cations for 2 h.

- - 1. *The selectivity of chelating agents towards metal cations on a mixed metal solution*

The selectivity of the chelating agents for metal ions in a mixed-ion solution was assessed using a 20 ppm mixture of Cd(II), Pb(II), and Cr(VI). The pH of the solution was adjusted to 3, and the extraction process was conducted over a period of 2 h.

- - 1. *Regeneration and re-use of the chelating agents*

After extracting metal cations, **L1@MCM-41** was immersed in 15 ml of 0.5 M HNO_3_ solution in conical flasks and stirred for 1 h at room temperature. The solution was centrifuged at 500 rpm, and the chelating agent was washed with 15 ml of deionized water and further centrifuged and separated. The regenerated chelating agent was dried in the vacuum oven at 60 ℃ for 4 h and employed for re-extraction.

The extraction efficiency of the metal cations by the chelating agents was determined using Inductively Coupled Plasma Atomic Emission Spectroscopy (ICP-AES), and the percentage extraction efficiency (% R) was calculated using the following equation:

%R = $\frac{\left( C_{i}-C_{f} \right)}{C_{i}}X 100$ (1)

The equilibrium extraction (qe) was calculated using the equation:

$q_{e}=\frac{\left( C_{i}-C_{f} \right) \times V}{M}$ (2)

where 𝑞_𝑒_​ is the amount of metal cations extracted from the aqueous solution (mg/g), ​𝐶_i_ and C_f_ are the initial and final concentrations of the metal cations before and after extraction, respectively (mg/L), V is the total volume of the metal salt solution (L), and M is the mass of the chelating agent used for extraction (g).


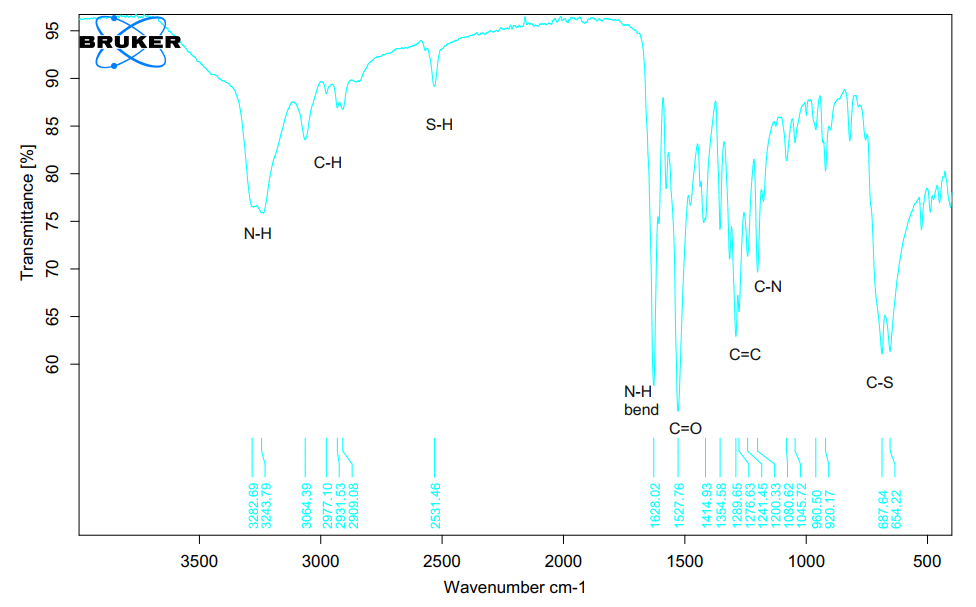


**Figure S1**. FT-IR of N^1^,N^3^-bis(2-mercaptoethyl)isophthalamide (**S1**).


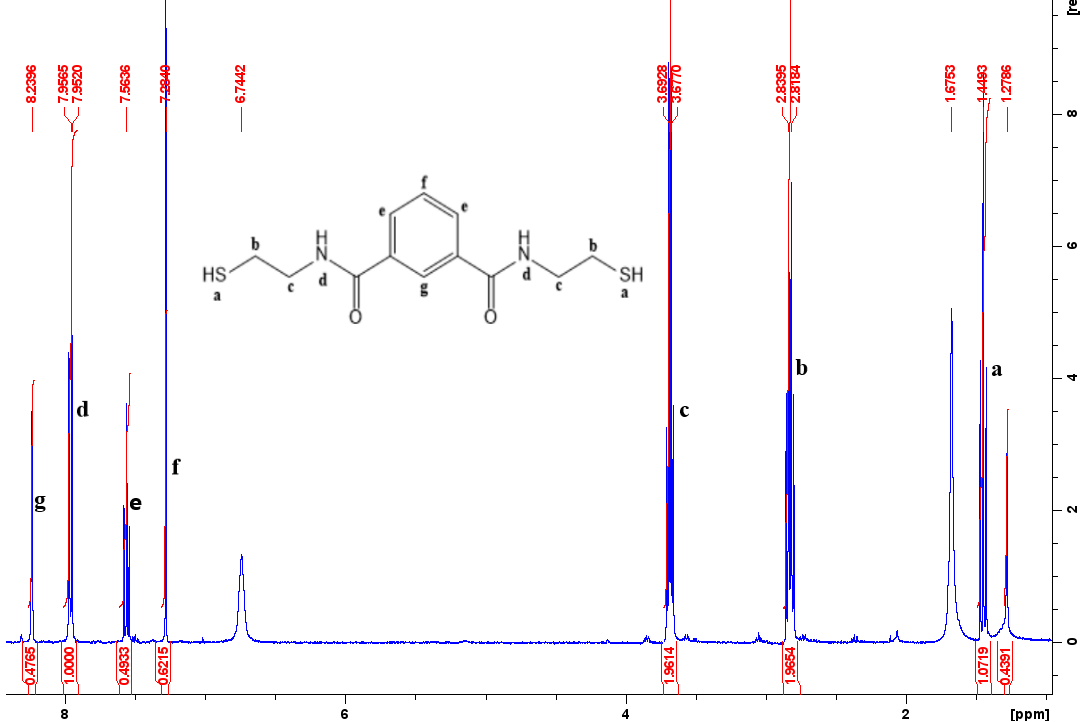


**Figure S2**. ^1^HNMR of N^1^,N^3^-bis(2-mercaptoethyl)isophthalamide (**S1**) in CDCl_3_.


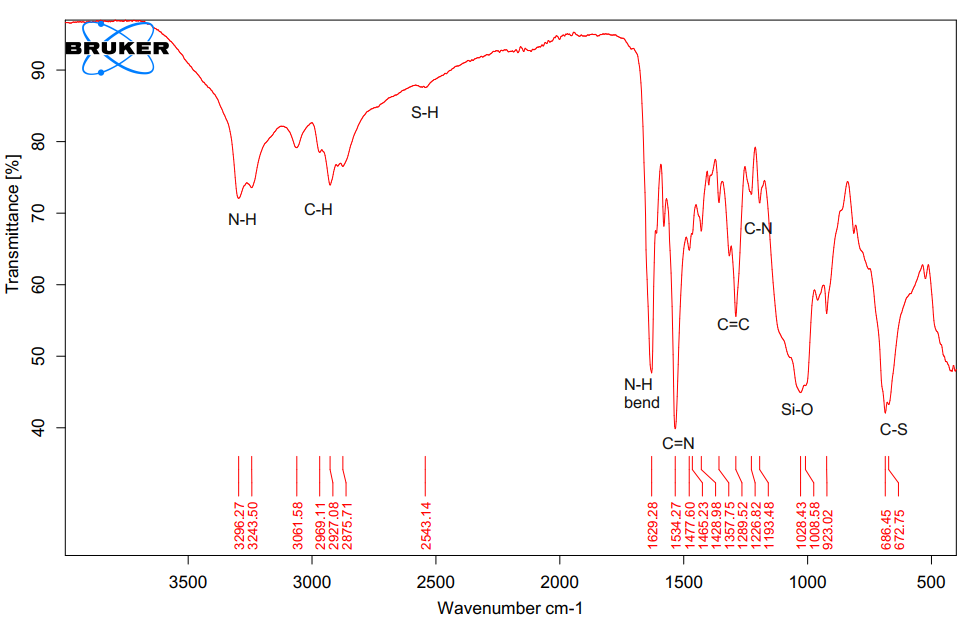


**Figure S3**. FT-IR of (1Z,3Z)-N^1^,N^3^-bis(2-mercaptoethyl)-N'^1^,N'^3^ bis(3(triethoxysilyl)propyl) isophthalimidamide (**L1**).


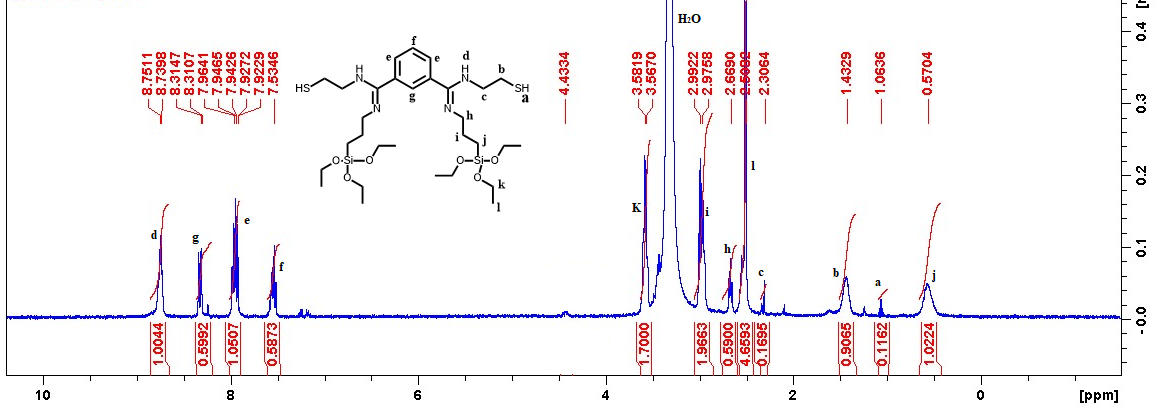
**Figure S4**. ^1^HNMR of (1Z,3Z)-N^1^,N^3^-bis(2-mercaptoethyl)-N'^1^,N'^3^ bis(3(triethoxysilyl)propyl) isophthalimidamide (**L1**) in CDC1_3_.


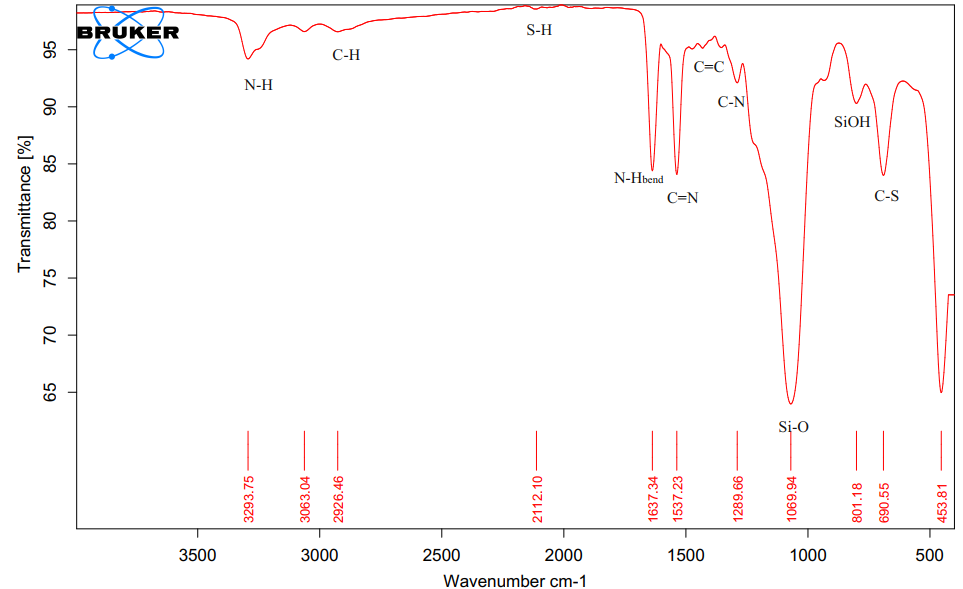
**Figure S5**. FT-IR of **L1@SBA-15**.


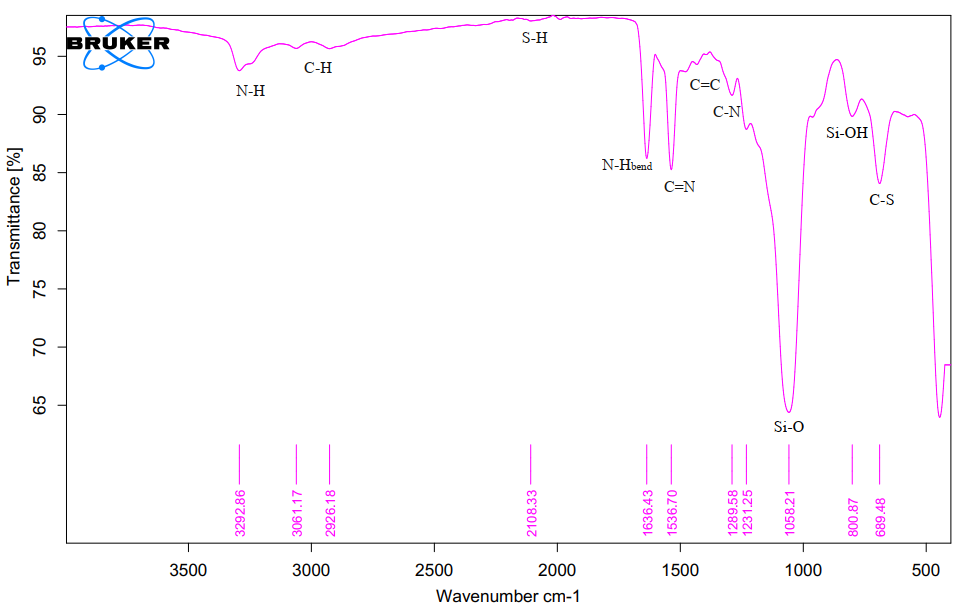
**Figure S6**. FT-IR of **L1@MCM-41**.

**Figure S7:** Average nanoparticle size of (**A**) **L1@SBA-15** and (**B**) **L1@MCM-41** measured using ImageJ software from TEM images.

**A**

**B**

**
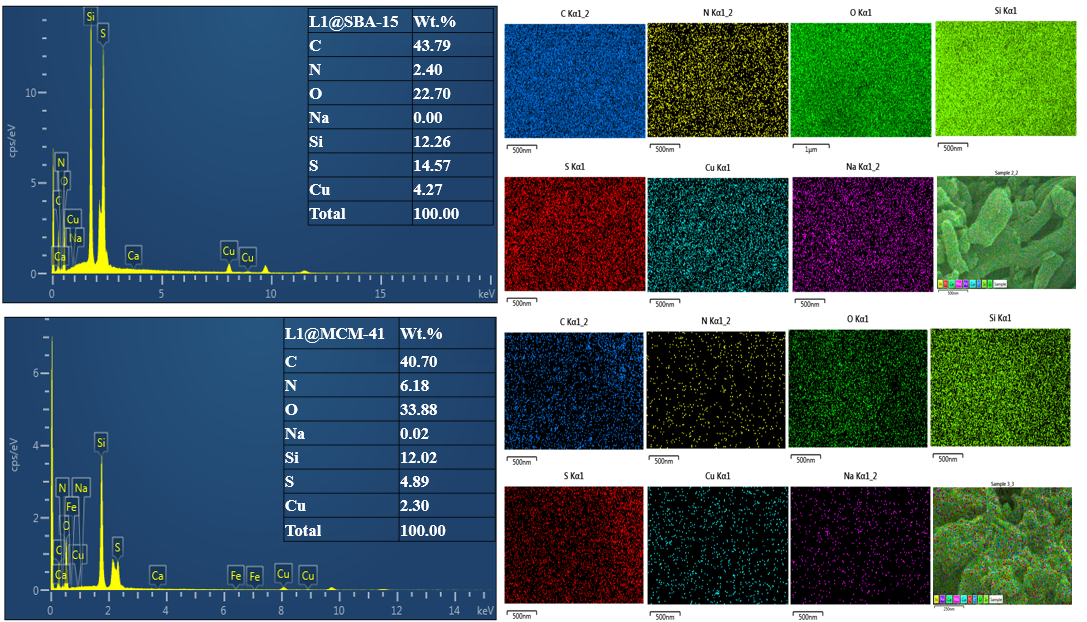
Figure S8.** Elemental composition and mapping of elements in the structure of **L1@SBA-15** and **L1@MCM-41** chelating agents.

**Figure S9:** Nitrogen adsorption-desorption isotherms and pore size distribution of (**A**) **L1@SBA-15** and (**B**) **L1@MCM-41**.


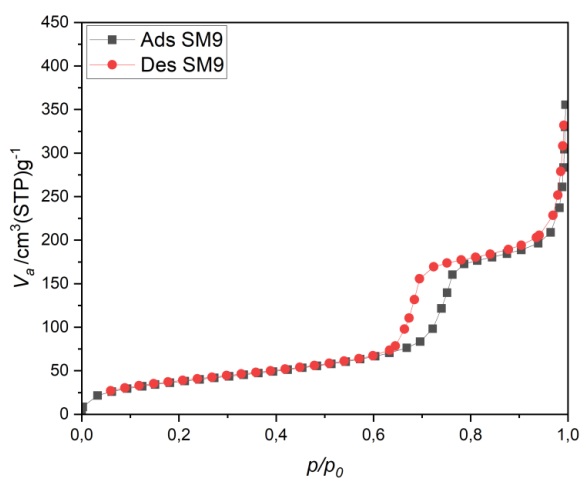

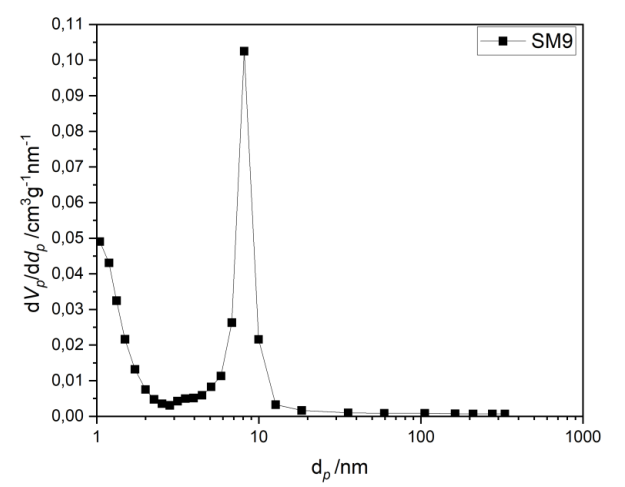

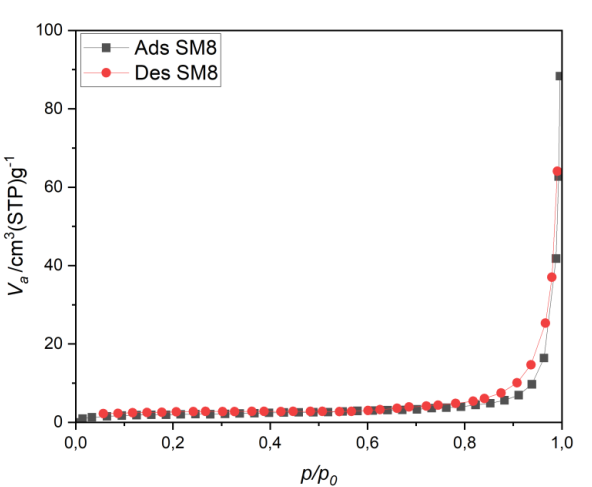

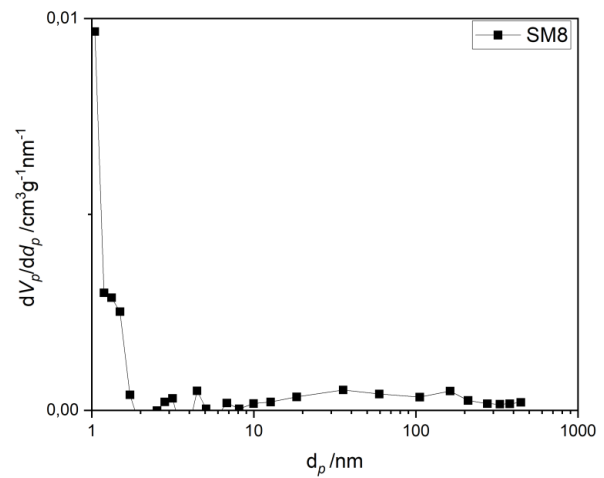


**A**

**B**


**Figure S10**. pXRD of **L1@SBA-15** and **L1@MCM-41**.

**Figure S11.** TGA/DTA-DSC curves of **L1@SBA-15** and **L1@MCM-41**.

**Figure S12:** Extraction of metal cations using **L1@MCM-41** fitted on (**A**) Langmuir, (**B**) Freundlich, and (**C**) Temkin adsorption isotherms.

**(A) L1@MCM-41 Langmuir isotherm**

**(B) L1@MCM-41 Freundlich isotherm**

**(C) L1@MCM-41 Temkin isotherm**

**Figure S13.** FT-IR of the chelating agents before and after extraction, showing the following band shifts; (**A**) [M(**L1@SBA-15**)]: *v*(N-H) at 3295 cm⁻¹, *v(*N-H) bending at 1637 cm⁻¹, *v*(C=N) at 1534 cm⁻¹, *v*(C-N) at 1295 cm⁻¹, and *v*(C-S) at 694 cm⁻¹ and (**B**) [M(**L1@MCM-41**)]: *v*(N-H) at 3299 cm⁻¹, *v*(N-H) bending at 1631 cm⁻¹, *v*(C=N) at 1532 cm⁻¹, *v*(C-N) at 1287 cm⁻¹, and *v*(C-S) at 681 cm⁻¹.

**A**

**B**

**Figure S14**. FT-IR spectra of ligand **L1** and its Cd_2_(**L1**)_2_ complex, illustrating the disappearance of the v_(S-H_) band at 2061 cm⁻¹ and the shifts of v(_N-H_), v(_N-H_) bending, v(_C-N_), and v(_C-S_) bands from 3299 cm⁻¹, 1643 cm⁻¹, 1229 cm⁻¹, and 679 cm⁻¹ to 3293 cm⁻¹, 1637 cm⁻¹, 1194 cm⁻¹, and 687 cm⁻¹, respectively, upon complexation.


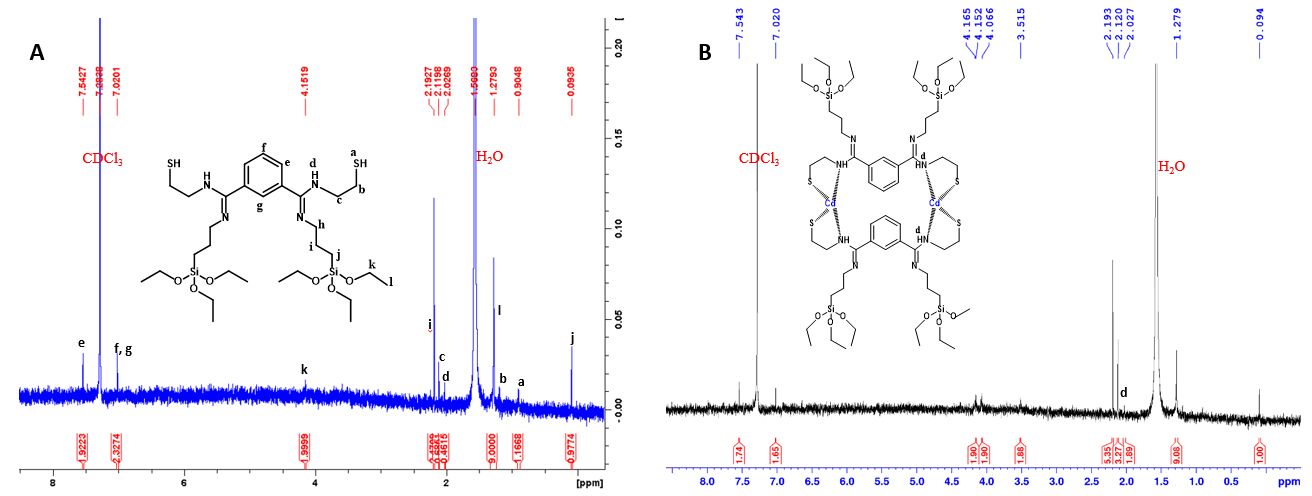


**Figure S15**. ^1^HNMR spectra of (**A**) ligand **L1** and (**B**) its Cd_2_(**L1**)_2_ complex in CDCl_3_, showing the HS-CH_2_ signal at 0.98 ppm and the H_2_C-NH signal at 2.026 ppm in **L1** prior to complexation. Upon complexation, the HS-CH_2_ signal disappears, and the H_2_C-NH signal shifts slightly to 2.027 ppm in Cd_2_(**L1**)_2_.

**Table S1**. Pseudo-first-order and pseudo-second-order adsorption kinetics models

| Adsorption kinetic model | Parameters |
| --- | --- |
| The pseudo-first-order kinetic model incorporates external and internal diffusion, as well as integrated adsorption (Pereao). The pseudo-first-order kinetic model assumes that the rate of adsorption is proportional to the number of available bonding sites (Kampalanonwat and Supaphol 2014). The pseudo-first-order kinetic model is also known as the Lagergren equation; it is expressed as follows:  ln (qe – qt) = ln qe – K_1_ (3) | Where q_e_ and q_t_: extraction amounts of heavy metal ions at equilibrium (mg/g) and time t (min), respectively. K_1_: rate constant of the pseudo-first-order kinetic model (min^-1^). The equilibrium extraction amount (q_e_) and rate constant (K_1_) values were determined as antilogarithmic values of the y-intercept and slope, respectively through plotting log (q_e_ – q_t_) versus t. |
| The adsorption process of the pseudo-second-order kinetic model is controlled by the chemisorption process. This model assumes that the rate of the adsorption process is proportional to the square of the unoccupied adsorption site number (Lasheen et al. 2012). The pseudo-second-order kinetic model can be expressed as follows:  $\frac{t}{q_{t}}=\frac{1}{K_{2}q_{e}^{2}}+\frac{t}{q_{e}}$(4) | Where K_2_: rate constant of the pseudo-second-order kinetic model (g/mg min). The values of equilibrium adsorption capacity (q_e_) and the rate constant (K_2_) were obtained from the plot of $\frac{t}{q_{t}}$ versus t as the inverse values of the slope and y-intercept, respectively. |

**Table S2:** Summary of kinetics parameters on the extraction of metal cations using **L1@MCM-41** and **L1@SBA-15** chelating agents.

|  |  | | Pseudo-first-order kinetic | | | | Pseudo-second-order kinetics | | | |
| --- | --- | --- | --- | --- | --- | --- | --- | --- | --- | --- |
| Chelating agents | Metal cations | q_e, exp_  (mg. g^1^) | K_1_  (h^-1^) | q_e, calc_  (mg. g^-1^) | | R^2^ | K_2_  (h^-1^) | q_e, calc_  (mg. g^-1^) | | R^2^ |
|  | Cd(II) | 14.9649 | 1.60605 | 2.6671×10^-4^ | 0.889 | | 9.7219 | 9.88044 | 0.97573 | |
| **L1@MCM-41** | Pb(II) | 14.9649 | 1.56260 | 2.5998×10^-4^ | 0.96039 | | 9.3984 | 9.99200 | 0.97348 | |
|  | Cr(VI) | 29.300 | 0.80727 | 20.08399 | 0.72471 | | 9.2980 | 33.5570 | 0.91017 | |
|  | Cd(II) | 14.9904 | 1.2757 | 5.7603×10^-3^ | 0.93344 | | 9.6815 | 9.91080 | 0.9754 | |
| **L1@SBA-15** | Pb(II) | 14.9834 | 1.2738 | 4.2983×10^-3^ | 0.8097 | | 9.1567 | 10.0674 | 0.97089 | |
|  | Cr(VI) | 16.2750 | 0.7739 | 8.58894 | 0.65701 | | 8.2726 | 19.4590 | 0.89411 | |

**Table S3**. Adsorption isotherms models.

| Adsorption isotherm model | Parameters |
| --- | --- |
| The Langmuir assumes irreversible and monolayer extraction. The following linear Langmuir equation was used to determine relevant Langmuir parameters:  $\frac{C_{e}}{q_{e}}=\frac{1}{K_{L}q_{m}}+\frac{C}{q_{m}}$ (5) | q_e_: Maximum extraction capacity at equilibrium (mg/g), C_e_: initial concentration of the metal cations(mg/L), q_m_: monolayer extraction capacity (mg/g), K_L_: Langmuir constant (L/mg). q_m_: inverse of the slope, K_L_ : intercept and slope values through a linear plot of $\frac{C_{e}}{q_{e}}$ versus C_e_. |
| Freundlich's model describes heterogeneous systems. The adsorption process can either be chemical or physical on non-uniform as well as uniform surfaces. The following linear equation was used to evaluate parameters:  log q_e_ = log k_F_ + $\frac{1}{n}$ log C_e_ (6) | K_F_: Freundlich equilibrium constant (mg/g) (L/mg)^1/n^, n: Freundlich constant that is related to the size of the extraction capacity. n and K_F_ were obtained from a plot of log q_e_ versus log C_e_. |
| The Temkin model assumes a non-linear interaction between adsorption energy and chelating agent surface. It assumes that as surface coverage increases, the adsorption energy reduces logarithmically. A linear form of the Temkin model can be expressed by the following equation:  $q_{e}=\left( \frac{RT}{B_{T}} \right) lnK_{T}+\left( \frac{RT}{B_{T}} \right) lnC_{e}$(7) | q_e_: Amount of metal cations extracted at equilibrium (mg/g), C_e_: initial concentration of metal cations(mg/L), B_T_: Temkin constant related to the heat of adsorption, K_T_: equilibrium binding constant corresponding to the maximum binding energy (L mg^−1^ ), R: gas constant (8.314 J mol^−1^ K^−1^ ). The constants K_T_ and B_T_ can be determined from the intercept and the slope of the linear plot of data q_e_ versus ln C_e_. |

**Table S4:** Summary of parameters of adsorption isotherms on the extraction of metal cations using **L1@MCM-41** and **L1@SBA-15** chelating agents.

|  |  | **L1@MCM-41** |  |  |
| --- | --- | --- | --- | --- |
|  | parameters | Cr(VI) | Cd(II) | Pb(II) |
| Langmuir | q_exp_(mg/g) | 10.8750 | 64.2750 | 74.9131 |
|  | q_m_(mg/g) | 6.133×10^-3^ | 497.512 | 4765.69 |
|  | K_L_(L/mg) | 5.465×10^-3^ | 1.524×10^-3^ | 1.552×10^-4^ |
|  | R^2^ | 0.27408 | 0.85501 | 0.46722 |
| Freundlich | K_F_(mg/g) | 1.605×10^-4^ | 0.61021 | 0.48254 |
|  | n | 0.80671 | 1.03936 | 0.99340 |
|  | R^2^ | 0.90923 | 0.99879 | 0.99999 |
| Temkin | B_T_(J/mol) | 841.103 | 120.685 | 105.865 |
|  | K_T_(L/g) | 6.617×10^-3^ | 0.01425 | 0.01244 |
|  | R^2^ | 0.64903 | 0.91029 | 0.88243 |
|  |  | **L1@SBA-15** |  |  |
|  | parameters | Cr(VI) | Cd(II) | Pb(II) |
| Langmuir | q_exp_(mg/g) | 12.0750 | 43.3500 | 74.5206 |
|  | q_m_(mg/g) | 2.88052 | 103.306 | 545.448 |
|  | K_L_(L/mg) | 9.772×10^-3^ | 8.158×10^-3^ | 1.229×10^-3^ |
|  | R^2^ | 0.54693 | 0.87684 | 0.40582 |
| Freundlich | K_F_(mg/g) | 1.239×10^-5^ | 1.14203 | 0.30077 |
|  | n | 0.93697 | 1.18200 | 0.94782 |
|  | R^2^ | 0.97374 | 0.97952 | 0.99892 |
| Temkin | B_T_(J/mol) | 563.910 | 170.508 | 105.737 |
|  | K_T_(L/g) | 7.807×10^-3^ | 0.02289 | 0.01188 |
|  | R^2^ | 0.85705 | 0.9657 | 0.88423 |

**Scheme S1.** Proposed mechanism for the extraction of metal cations using chelating agents.

**Scheme S2**. Synthesis of [**Cd_2_(L1)_2_**] complex and proposed structure.

**References**

Dang Son BH, Quang Mai V, Xuan Du D, et al (2016) A Study on Astrazon Black AFDL Dye Adsorption onto Vietnamese Diatomite. Journal of Chemistry 2016:1–11. https://doi.org/10.1155/2016/8685437

El-Sheikh AH, Nofal FS, Shtaiwi MH (2019) Adsorption and magnetic solid-phase extraction of cadmium and lead using magnetite modified with schiff bases. Journal of Environmental Chemical Engineering 7:103229. https://doi.org/10.1016/j.jece.2019.103229

Kampalanonwat P, Supaphol P (2014) The Study of Competitive Adsorption of Heavy Metal Ions from Aqueous Solution by Aminated Polyacrylonitrile Nanofiber Mats. Energy Procedia 56:142–151. https://doi.org/10.1016/j.egypro.2014.07.142

Lasheen MR, Ammar NS, Ibrahim HS (2012) Adsorption/desorption of Cd(II), Cu(II) and Pb(II) using chemically modified orange peel: Equilibrium and kinetic studies. Solid State Sciences 14:202–210. https://doi.org/10.1016/j.solidstatesciences.2011.11.029

Pereao OK Functionalisation of electrospun nanofibre for lanthanide ion adsorption from aqueous solution

Shtaiwi MH, Tahboub DM, El-Sheikh AH, Al-Hashimi NN (2020) Magnetic solid-phase extraction of metal ions: Comparison of adding Schiff base to the extraction medium vs. magnetite modification with Schiff base. Journal of Environmental Chemical Engineering 8:103675. https://doi.org/10.1016/j.jece.2020.103675

Zhang M, Zhang S, Sun L, et al (2020) One-step synthesis of 2-mercaptobenzothiazole functionalized magnetic Fe3O4 and its application for the removal of heavy metals. Journal of the Taiwan Institute of Chemical Engineers 113:264–272. https://doi.org/10.1016/j.jtice.2020.08.017
